# DNA Polymerases λ and β: The Double-Edged Swords of DNA Repair

**DOI:** 10.3390/genes7090057

**Published:** 2016-08-31

**Authors:** Elisa Mentegari, Miroslava Kissova, Laura Bavagnoli, Giovanni Maga, Emmanuele Crespan

**Affiliations:** Institute of Molecular Genetics, IGM-CNR, via Abbiategrasso 207, 27100 Pavia, Italy; elisa.mentegari01@universitadipavia.it (E.M.); miroslava.kissova01@universitadipavia.it (M.K.); bavagnoli@igm.cnr.it (L.B.)

**Keywords:** DNA polymerases, DNA repair, translesion synthesis, cancer chemotherapy, mutagenesis

## Abstract

DNA is constantly exposed to both endogenous and exogenous damages. More than 10,000 DNA modifications are induced every day in each cell’s genome. Maintenance of the integrity of the genome is accomplished by several DNA repair systems. The core enzymes for these pathways are the DNA polymerases. Out of 17 DNA polymerases present in a mammalian cell, at least 13 are specifically devoted to DNA repair and are often acting in different pathways. DNA polymerases β and λ are involved in base excision repair of modified DNA bases and translesion synthesis past DNA lesions. Polymerase λ also participates in non-homologous end joining of DNA double-strand breaks. However, recent data have revealed that, depending on their relative levels, the cell cycle phase, the ratio between deoxy- and ribo-nucleotide pools and the interaction with particular auxiliary proteins, the repair reactions carried out by these enzymes can be an important source of genetic instability, owing to repair mistakes. This review summarizes the most recent results on the ambivalent properties of these enzymes in limiting or promoting genetic instability in mammalian cells, as well as their potential use as targets for anticancer chemotherapy.

## 1. Introduction

DNA polymerases (Pols) β and λ belong to the Pol family X. In mammalian cells, the X family comprises four members: Pol β, λ, μ and terminal deoxynucleotidyl transferase (TdT). All of the members possess a highly homologous C-terminal catalytic or β-like domain. In addition, Pol λ, μ and TdT contain an extended N-terminus, with additional domains. While the functions of Pol μ and TdT seem to be restricted to specialized forms of DNA double-strand break repair, such as non-homologous end joining (NHEJ) and V(D)J recombination, Pol β and λ play more diversified roles, participating in different DNA repair pathways. Because of their partially overlapping roles, Pol β and λ are regulated along the cell cycle, through mainly post-translational modifications [1,2,3,4]. In addition, their function is also under the control of cell cycle checkpoints [5,6]. Since these Pols are involved in the tolerance of various kinds of DNA damages, including those caused by anticancer chemo- or radio-therapy, and their overexpression may lead to genetic instability, they are being regarded as attractive targets for cancer chemotherapy. Below, we will provide a summary of the roles of Pol β and λ in the various repair pathways, their regulation and the state of the art in the development of specific inhibitors for these enzymes.

## 2. Pol β and λ in the Base Excision Repair Pathway

Pol β and λ use their polymerase and 5′-deoxyribose 5′-phosphate (dRP)-lyase activities during single-nucleotide base excision repair (BER) [7]. 

The BER pathway contributes to the maintenance of genome integrity, since it repairs DNA lesions caused by alkylation, oxidation, depurination/depyrimidination and deamination [8] that, if not removed, could cause mutations by mispairing or lead to strand breaks during replication. Enzymes acting during BER excise the damaged nucleotide and replace it with the correct one [9].

In eukaryotes, the pathway is divided into short-patch (SP) BER, devoted to the replacement of single damaged nucleotides, and long-patch (LP) BER to repair two or more damaged nucleotides. Pol β is the solely Pol involved in SP BER, while in LP BER, it is involved in the incorporation of the first nucleotide, whereas the elongation step is carried out by replicative Pols [10]. Both pathways start with the damaged base being recognized; then, a DNA glycosylase hydrolizes the N-glycosidic bond removing the base from the sugar-phosphate backbone. At this point, the AP (abasic or apurinic/apyrimidinic) site generated in this way is further processed by apurinic/apyrimidinic endonuclease 1 (APE1), the main 5′ AP endonuclease in human cells, which cleaves the phosphodiester bond 5′ to the AP site, generating a 5′dRP and a 3′-OH. The 5′dRP is removed by Pol β, as it possesses intrinsic dRP-lyase activity, leaving a phosphate at the 5′-end. Subsequently, Pol β can fill the gap, and finally, ligase III in complex with its accessory protein XRCC1 ligates the nicked DNA in the SP BER [10,11]. Experiments performed on mouse cells demonstrated that ligase III, which possesses nuclear and mitochondrial forms, is fundamental for the maintenance of the mitochondrial DNA (mtDNA) integrity, while it is not required in nuclear BER [12,13]. In LP BER, the final ligation step is instead performed by ligase I [11]. Like Pol β, also Pol λ is involved in BER: while Pol β is the main enzyme for BER, Pol λ involvement in BER is supported by the observation that it can substitute Pol β in BER reactions in vitro [8]. Indeed, it has been shown that Pol λ is the preferred Pol involved in the specialized BER, which removes 7,8-dihydro-8-oxoguanine (8-oxo-G):A mispairs, initiated by the glycosylase MUTYH [14] (see also below).

## 3. Pol β and λ in the Translesion Synthesis Pathway

DNA integrity is fundamental for the inheritance of complete and correct genetic information. Cells are exposed not only to exogenous attacks, such as ionizing radiation, ultraviolet light and chemical agents, but they also have to cope with endogenous mechanisms generating reactive metabolites that are threatening for DNA. Despite multiple repair pathways evolved in order to correct damages occurring in DNA, other mechanisms are necessary to tolerate DNA lesions without actually repairing them. DNA damage tolerance processes are important in promoting cell survival and, in some cases, contribute to the generation of mutations [15]. During the S phase, when the replication machinery encounters a lesion along DNA, replicative Pols are unable to bypass it and to incorporate the right nucleotide opposite the damaged site, leading to fork stalling. In such a situation, the translesion synthesis (TLS) mechanism is activated.

TLS is one of the major damage tolerance systems in which specialized polymerases, known as TLS Pols, substitute for replicative Pols in copying across DNA lesions during replication [8]. TLS Pols are able to use damaged DNA bases as a template and to insert nucleotides opposite them. 

Two non-mutually exclusive models for lesion bypass by TLS Pols have been proposed: the polymerase-switching model and the gap-filling model. At the replication fork, where replicative Pols are acting, a switch occurs in the presence of DNA damage through protein-protein interactions, so that replicative Pols are substituted by TLS Pols. After lesion bypass with relative accuracy, an additional switch takes place, and the replicative Pol is restored in order to continue accurate DNA synthesis [15]. It has also been proposed that TLS takes place outside replication forks, in a gap-filling model. In this scenario the replication machinery leaves a single-strand DNA (ssDNA) gap opposite a DNA lesion because of suppressing events that occur downstream of the damage, leaving to TLS Pols the role of filling the gap [15].

TLS Pols are present in all three domains of life, and they mainly belong to the Y family of DNA Pols; however, also Pol β and λ play a role in specialized forms of TLS, even if it is not their primary task [16].

### 3.1. Bypass of the 7,8-Dihydro-8-Oxoguanine (8-oxo-G) Lesion

Oxidative lesions are one of the most frequently-observed base modifications; they derive from the action of reactive oxygen species (ROS) [17]. Hydroxyl radicals, in particular, can generate adducts at diverse positions of purines, since they add to the double bonds of DNA bases. The C_8_-OH adducts of guanine, such as 7-hydro-8-hydroxyguanine and 7,8-dihydro-8-oxoguanine (8-oxo-G), and the C_2_-OH adduct of adenine are the most studied oxidative lesions [17]. 8-oxo-G is a particularly relevant lesion because it is widely present in DNA (10^3^ to 10^4^ per cell per day) [18] and because of its well-established mutagenic potential in bacterial and mammalian cells. It is a miscoding lesion that can generate G:C to T:A transversions; it accumulates with age, mainly in the mitochondrial genome, and it is involved in different types of tumors and neurodegenerative diseases (e.g., Parkinson and Alzheimer diseases) [17].

8-oxo-G can be bypassed by replicative Pol α, δ and ξ in an error-prone manner since, in many cases, instead of inserting the correct cytosine (C) opposite to the damage, they incorporate an adenine (A) [16]. In order to face this threatening damage, prokaryotic and eukaryotic cells evolved two different BER systems: an 8-oxo-G DNA glycosylase 1 (OGG1)-dependent pathway and a MUTHY-dependent pathway [18]. In all tissues, the OGG1 initiates the short patch BER recognizing the damaged G when paired with a C, catalyzing the removal of 8-oxo-G. The AP site generated in this way is a substrate of APE1, then DNA Pol β fills the gap, and DNA ligase III/XRCC1 complex performs the ligation step [16,17]. In replicating tissues, when the replication machinery encounters the 8-oxo-G, it often incorporates an A instead of a C; the glycosylase MUTYH has the ability to recognize the mispair, but excides the A. At this point, APE1 incises the DNA, then DNA Pol λ with the help of Proliferating cell nuclear antigen (PCNA) and replication protein A (RP-A) incorporates the correct C opposite the 8-oxo-G left on the template strand within the gap [14]. The newly-formed C:8-oxo-G pair can subsequently become the substrate of the OGG1-dependent short patch BER [16]. 

Since both Pol λ and β have a role in BER, there should be a mechanism allowing the selection of one or the other to repair the 8-oxo-G lesion. Experiments suggested that PCNA and RP-A have a role in this discriminatory mechanism, recruiting Pol λ instead of Pol β towards the 8-oxo-G lesion facing a window gap. Pol λ is the most efficient in the MUTYH-initiated pathway, ensuring error-free TLS with correct Deoxycytidine triphosphate (dCTP) incorporation opposite 8-oxo-G; its fidelity is increased by the association with PCNA and RP-A, ensuring a 750-fold preference for dCTP incorporation opposite to 8-oxoG on 1-nt gap with respect to Deoxyadenosine triphosphate (dATP) incorporation. The other main polymerase involved in BER, Pol β can substitute for Pol λ, but at the expense of a reduced fidelity, leading to frequent misincorporation of dATP opposite 8-oxo-G (in 20% to 30% of cases). In fact, Pol β is 145-fold less efficient than Pol λ in bypassing 8-oxo-G damage on 1-nt gaps inserting the correct dCTP [18].

### 3.2. Bypass of Abasic Sites and the 2-Deoxyribonolactone Lesion

Reactive oxygen species (ROS) have also the ability to cause the accumulation of oxidized AP sites [19]. Among the oxidized AP sites produced by oxidative stress, the C1’-oxidized abasic site 2-deoxyribonolactone (L) is a frequently-encountered lesion, representing about 10% of total 2-deoxyribose oxidation [20]. L can be caused also by long wave UV irradiation, organometallic oxidants and by antitumor drugs, such as neocarzinostatin and the enediyne antibiotic C-1027 [21]. The presence of L in the DNA strand, on the other hand, can be particularly dangerous during the S phase, since it can lead to the arrest of the replication fork.

AP sites are usually handled by the BER pathway, where an AP endonuclease, mainly APE1 in mammalian cells, incises AP sites, thus generating ssDNA breaks with 3′-OH and 5′-deoxyribose-5′-phosphate (5′-dRp) termini. Pol β, through its 5′-dRp-lyase activity residing in its 8-kDa domain, can remove the 5′-dRp residue [22,23]. Pol λ possesses a homologous 8-kDa domain, which also allows elimination of 5′-dRp [24]. Both Pol λ and β can bypass non-oxidized AP sites, but with diverse mechanisms. In most cases, Pol λ skips the lesion and gives rise to a -1 frameshift deletion, while Pol β generally incorporates a dATP opposite the AP site [21,25]. Moreover, Pol λ is more efficient than Pol β in performing TLS when an AP site is present, especially when the concentration of nucleotides is low [25].

On the contrary, when L lesion or chemically-reduced AP sites are present, Pol β cannot excise the modified sugar. Therefore, LP BER is activated in order to process such lesions. However, during unsuccessful attempts to repair L through SP BER, when Pol β attacks the 5′-dRp residue through the active site of its N-terminal lysine 72 (K72), it becomes covalently trapped on DNA via the formation of an amide bond with K72, resulting in the formation of DNA-protein cross-links (DPC) [22,23,24,26,27].

In in vitro experiments, performed using either Mn^2+^ or Mg^2+^ as the cofactor, Pol β exhibited the ability of bypassing L, and, its capacity of performing TLS over such a lesion, was enhanced in the presence of the auxiliary protein PCNA. Pol β mainly incorporates dATP opposite L and, to a minor extent, dCTP. Thus, L bypass, similarly to the case of the normal AP site, is most of the times mutagenic. On the other hand, similar experiments revealed that Pol λ is unable to bypass L damage, even when nucleotide concentrations were high [21].

### 3.3. Pol δ-Interacting Protein 2 as an Auxiliary Factor for Pol λ during TLS

In the presence of a DNA lesion, a switch between replicative Pols and TLS Pols allows lesion bypass can occur. DNA Pol δ-interacting protein 2 (PolDIP2), also known as Pol δ interacting protein 38 (PDIP38), is a protein of 368 aa that makes contact with the p50 subunit of Pol δ and with PCNA [28], the processivity clamp whose ubiquitination seems to favor the access of TLS Pols to the lesioned DNA site [29]. In effect, in the presence of PolDIP2, Pol δ increases its affinity for PCNA by about two-fold [30].

PolDIP2 also physically interacts with TLS Pol η, ζ and Rev1, even if the physiological meaning of this event is still required to be fully elucidated. Indeed, PolDIP2 can associate to the ubiquitin-binding zinc finger domain of Pol η, the domain that mediates the interaction of Pol η with ubiquitinated PCNA. An intriguing possibility is that PolDIP2 might have a role in the TLS pathway, contributing to the switch between Pol η and TLS Pols [30].

While PolDIP2 does not seem to stimulate either Pol β or Pol ι, it has been found to physically interact with Pol λ, the main actor in bypassing faithfully the 8-oxo-G lesion by inserting in most cases the right cytidine. Pol λ forms a complex with PolDIP2 through its catalytic domain. Experiments demonstrated that PolDIP2 association with Pol λ, as well as to Pol η, positively regulates their ability to perform correct 8-oxo-G bypass. PolDIP2 enhances both the processivity and catalytic activity of Pol λ and η, thus favoring a speeding up of the bypass process, not only of 8-oxo-G damage, but of other DNA lesions, as well, such as abasic sites and cyclobutane thymine dimers. In particular, PolDIP2 favors the switch from Pol δ to Pol λ in TLS of the 8-oxo-G lesion [30].

Moreover, silencing of PolDIP2 in mouse embryonic fibroblasts (MEFs) results in increased sensitivity of cells to oxidative agents, an effect very similar to the one displayed by Pol λ-null cells. When PolDIP2 was silenced in Pol λ^−/−^ fibroblasts, the sensitivity further increases, thus suggesting that cells need both Pol λ and PolDIP2 for effective DNA damage response. [30]. Interestingly, PolDIP2 has been also shown to activate the intracellular oxidase NADPH oxidase 4 (Nox4), thus increasing endogenous ROS levels. Since this protein shuttles from the cytoplasm to the nucleus in response to proliferative stimuli, it is intriguing to speculate that during replication, when the risk of incurring mutations due to oxidized bases is higher, PolDIP2 stops stimulating Nox4 and aids TLS Pols in bypassing oxidative DNA damages caused by endogenous ROS.

## 4. Pol β and λ and the Incorporation of Ribonucleotides in the Genome

The selectivity of the incorporation of dNTPs is from 10-fold to 10^6^-fold greater with respect to ribonucleotide monophosphates (rNMPs) incorporation, depending on the identity of the polymerase, on the base examined and on rNMP:Deoxynucleoside Monophosphate (dNMP) ratio [31]. However, in spite of their specificity, replicative Pols discriminate dNMPs imperfectly, and so, they can frequently incorporate rNMPs during DNA replication since in mammalian cells, as well as in yeast cells, rNMPs’ concentration is 10- to 100-fold higher than dNMPs’ concentration. [32,33].

Lacking the reactive hydroxyl group in position 2′ of the ribose ring, DNA is more stable and resistant to cleavage than RNA [31]. Therefore, for the cell, it is important to remove rNMPs in order to preserve the integrity of the genome. Indeed, most DNA Pols evolved to avoid incorporation of rNMPs during DNA synthesis, as they could lead to spontaneous strand breaks and stalling of Pols at the replication fork [32]. Most misincorporated rNTPs are removed through the RNaseH2-initiated ribonucleotide excision repair (RER) pathway [34].

Besides replication, rNMPs can be inserted in the genome by Pols also during reparative pathways. Until now, 17 Pols, including cytidyl-transferase Rev1 and telomerase, are known to be present in human cells, many of which participate in DNA repair pathways [16].

Reparative Pols are low-fidelity Pols that do not possess 3′ to 5′ exonuclease activity, so they cannot proofread rNMPs erroneously inserted as replicative Pols do. For high-fidelity Pols, the range of sugar selectivity is from 500 to 4,400,000, while for reparative low-fidelity Pols, the values range is between 1.3 and 50,000. This difference is probably ascribed to the overall flexibility and arrangement of their active sites [35].

Several studies have shown that family X Pols can incorporate rNMPs during the synthesis of undamaged DNA, but with varying sugar selectivity. Pol μ displays the lowest discrimination capability, in the range of 1- to 10-fold preference for dNMPs over rNMPs incorporation, thus possessing both DNA and RNA polymerase activities [36]. Pol μ can incorporate rNMPs and dNMPs with similar efficiency, because it possesses a glycine residue at the predicted “steric gate” position [37] where, instead, Pol β and λ have Tyr or Phe residues, thus achieving a higher sugar selection [35]. It has been shown that Pol λ has a sugar selectivity of 5100 to 7500 and Pol β of 1690 to 3200 for incorporation of rCMP opposite a guanine, depending on the structure of the template [38], in agreement with similar results [35,39]. These findings suggest that Pol β and λ can incorporate rNMPs opposite undamaged DNA bases [38]. Moreover, examining the impact of rNMPs’ incorporation opposite the 8-oxo-G lesion, Pol λ displayed the ability to bypass such a lesion on a 1-nt gap template incorporating the correct dCMP in the majority of cases. Pol β, on the other hand, having a lower selectivity for rNMPs, can bypass the lesion also inserting rCMP (wrong sugar/right base), but at least excluding in most cases rAMP (wrong sugar/wrong base) [38]. Incorporation of rNMPs opposite an 8-oxo-G lesion has also been shown to negatively impact the subsequent action of the glycosylases OGG1 and MUTYH, thus substantially delaying BER [38,40].

Thus, Pol β can be a source of rNMPs’ incorporation into genomic DNA, both during BER (that is opposite normal DNA bases) and during the bypass of 8-oxo-G. Pol β is the major Pol expressed in post-mitotic neurons, which are cells with dNTP levels markedly lower than rNTP levels, with a poor expression of RNaseH2 and that undergo severe oxidative stress. Therefore, post-mitotic neurons ability to use rNMPs may have physiological relevance in enhancing the deleterious effects of DNA oxidation in the brain [38].

## 5. Pol β and λ in Specialized Forms of DNA Double Stand Break Repair

Double-strand breaks (DSBs) represent the most dangerous damages occurring in DNA since they can lead to cell death if left unrepaired or cause chromosomal rearrangements if misrepaired. DSBs can result from endogenous sources, such as ROS, which can alter in different ways DNA bases, or they can arise from programmed processes, including V(D)J recombination and class switch recombination (CSR). Moreover, also, exogenous sources, like IR and ultraviolet (UV) light, can induce DNA DSBs [41].

Cells have evolved two main general mechanisms to face these genotoxic lesions, homologous recombination (HR) and non-homologous and joining (NHEJ) [41]. HR acts exclusively during the S phase, while NHEJ, the main DSB repair pathway in higher organisms, acts throughout the cell cycle [8]. The “classical” NHEJ starts with binding of the Ku70/80 heterodimer to the ends of the broken double-stranded DNA molecule, a step that allows successive binding of NHEJ factors. Pol μ and λ participate in the NHEJ pathway. They can bind to Ku:DNA complexes through their N-terminal breast cancer carboxy-terminal (BRCT) domains [42], providing their gap-filling activity before the final ligation step, which is performed by the XRCC4-ligase IV complex. Pol λ tends to fill gaps with ends that have partially complementary overhangs, while Pol μ can synthesize DNA without the presence of complementarity between the primer and template strand [43].

Besides classical NHEJ, an alternative Ku-independent and ligase IV-independent NHEJ pathway exists. It has been proposed that this alternative end-joining (A-EJ) pathway proceeds through microhomology-mediated end joining (MMEJ) [41,42,43]. It seems that in MMEJ, terminal microhomology (MH) can substitute for the presence of Ku protein [42]. This mechanism relies on MH regions (five to 25 nucleotides) that anneal to form a synaptic complex causing the formation of gaps on both DNA strands. These gaps are subsequently filled by Pols and finally ligated by DNA ligase 3 (Lig3) or DNA ligase 1 (Lig1) [41,42,43]. 

Evidence supports the involvement of Pol λ in MMEJ. When DSBs occur, nucleases create 3′ssDNA overhangs with terminal MH. The idea is that Pol λ promotes the creation of stable synapsis at MH regions, with the formation of long DNA gaps on both strands. The elongation step follows, during which the 9-1-1 complex increases the processivity of Pol λ, which makes contact with the 5′-phosphate of the terminal downstream nucleotide when the gap size reaches 1 nt, thus ensuring precise gap-filling. Finally, Lig1 seals the nick [43]. This reaction is also stimulated by the flap endonuclease 1 (FEN1) [43].

In vitro experiments demonstrated that Pol β is not able to promote annealing and elongation of long ssDNA 3′ overhangs possessing a single short MH region. On the other hand, it was capable of promoting the annealing and elongation of short (five nucleotides) 3′ ssDNA overhangs, even more efficiently than pol λ, in sequences containing CAG triplet repeats. Moreover, on such substrates, Pol β leads to the expansion of CAG triplets [43]. This observation, along with the fact that Pol β is the most highly expressed Pol in post-mitotic neurons, may suggest a role of MMEJ in the CAG repeats’ expansion linked to neurodegenerative diseases, such as the Huntington disease.

## 6. Pol β and λ Roles in Genetic Instability

Pols have an extremely important role in repairing DNA damage, protecting the cells. In fact, the damage, if not repaired, can lead to mutagenesis. However, unscheduled activation of repair Pols or alteration of their levels, can be detrimental for the cell, leading to genetic instability. For this reason, repair Pols must be tightly regulated. In recent years, further details about the regulation of Pol β and λ and their relationships with cell cycle checkpoints have emerged.

### 6.1. Pol λ

As summarized above, Pol λ plays a fundamental role during non-homologous end joining (NHEJ) and the bypass of DNA lesions. Some of these lesions, such as AP sites or oxidized bases, can slow down or even block replication fork progression [17,44]. As a consequence, the S phase checkpoint, relying on the ATR protein kinase pathway, is activated [6], which is responsible for cell survival in the presence of a stalled replication fork [45]. ATR activation leads to the phosphorylation and activation of the checkpoint kinase 1 (Chk1), which initiates a cascade of phosphorylation events that ultimately delays S phase progression [46] and activates the recruitment of DNA repair factors. Zucca et al. demonstrated that the downregulation of Pol λ resulted in the activation of the ATR/Chk1 pathway [5]. Cells permanently silenced for Pol λ accumulated replication stress, as evidenced by increased γH2AX histone foci, and showed phosphorylation of ATR and Chk1. Inhibition of Pol λ and Chk1 function resulted in cell lethality. One possible explanation is that, in the presence of Pol λ, oxidized bases can be efficiently bypassed [16]. However, in its absence, the accumulation of oxidized bases causes the block of the replication fork, slowing the repair of DNA damage and accumulating SS breaks. This causes the activation of the ATR-Chk1 pathway repair leading to the delay of the S phase of the cell cycle. These results highlighted the role of Pol λ in replication fork stability [5]. DNA Pol λ stability is also regulated during cell cycle by phosphorylation. Frouin et al. demonstrated that Pol λ interacts in the late S and G2 phases with Cdk2 in vivo, and it is phosphorylated by the Cdk2/cyclin A complex in vitro at Ser167, Ser17, Ser230 and Thr553 [3]. Markkanen et al. demonstrated that phosphorylation of Pol λ promotes its placement to 8-oxo-G lesions on chromatin, while Pol λ that is not phosphorylated and, as a consequence, is not involved in DNA repair is ubiquitinated by E3 ubiquitin ligase Mule and subsequently degraded via the ubiquitin-proteasome pathway [4]. The regulation by phosphorylation/ubiquitination probably allows Pol λ to properly repair the DNA damage during the S phase of the cell cycle.

In a large study [47], Pol λ has been found overexpressed in the 24% of various human solid tumors. Moreover, a cancer-related variant of Pol λ, the R438W mutant, was described, having low fidelity, impaired NHEJ capability and inducing genomic instability. These results highlight a role of Pol λ deregulation/mutation in promoting tumorigenesis [48,49].

### 6.2. Pol β

Pol β is the major polymerase involved in BER, its levels are regulated mainly through ubiquitination by the E3 ubiquitin ligases Mule and CHIP. Ubiquitination leads to Pol β degradation. The Mule inhibitor protein ARF and the human ubiquitin-specific protease 47 (USP47) deubiquitinating enzyme counteract this effect during DNA damage response, ensuring the maintenance of balanced levels of Pol β [1,2]. However, Fang et al. demonstrated that the XRCC1/Pol β complex formation prevents the ubiquitination and degradation of Pol β, which is otherwise ubiquitinated on Lys206 and Lys244 and targeted for proteasome-mediated degradation. The authors proposed that Pol β stability depends on the binding to XRCC1. Such a mechanism is used for DNA repair pathway choice, depending on the requirement of Pol β for the repair of specific damage (as in BER), while XRCC1, which is stable also as a monomer, is involved also in Pol β-independent repair pathways, such as NHEJ and NER [50].

Literature data demonstrated that 30% of tumors in human express several Pol β variants [51,52], and about 48% are characterized by aminoacidic substitutions [53,54]. The most common tumor-associated variants of Pol β are listed in Table 1.

As described above, the DNA substrate for Pol β is a single-nucleotide gap generated by the excision of a damaged base [11]. Ray. et al. reviewed that the variants reported can lead to genomic instability in several ways: the Pol β variant could misincorporate nucleotides in the gap, as is the case of Pol β variant K289M [55], leading to a mutagenesis process; the Pol β variant with slow or no polymerase activity (as is the case of the G231D and E295K variants, respectively [51]) does not insert any nucleotides in the gap, leading to the accumulation of double-strand breaks; the failure to remove the group from a dRP by the Pol β variant with slow lyase activity (L22P variant) [51] could also result in genomic instability [62]. 

Overexpression of Pol β is present in 30% of tumors, mostly solid tumors (gastric, uterine, prostate, thyroid and ovarian cancer) [47] and in chronic myeloid leukemia [63]. Bergoglio et al. demonstrated that overexpression of Pol β by only two-fold in cells is enough to promote genome instability, suggesting that Pol β regulation has a key role in vivo [64]. It has been demonstrated that overexpression of Pol β improved the mutator phenotype because of the genotoxic effects of oxidized damages [65]. Moreover, the alteration of Pol β expression in irradiated cells strengthened the genetic changes associated with a malignant phenotype [66].

Overexpression of Pol β could also cause genome instability, probably interfering with normal cellular processes. Starcevic et al. demonstrated that Pol β interacts with TRF2 protein [53], a telomeric DNA binding protein that has an important role in the maintenance of telomeres [67]. The authors supposed that overexpression of Pol β could sequestrate TRF2, causing the telomeres’ ends’ fusion, leading to chromosomal instability [53]. Polymerase β plays an important role in repairing DNA damage also during meiosis (Prophase I), maintaining genomic stability [56]. 

Trinucleotides repeat (TNR) instability is a feature of several neurological diseases, including Huntington disease (HD) and myotonic dystrophy 1 (MD1). Many studies linked the TNR expansion in somatic cells to erroneous DNA repair involving BER, nucleotide excision repair (NER) and mismatch repair (MMR). For a comprehensive review, see Goula and Merienne, 2013 [68]. Mounting evidence supports a crucial role of Pol β modulation by different BER factors in mediating somatic TNR expansion [69]. Moreover, recent findings showed that the MMR protein MutSβ physically interacts at the (CAG)n or (CTG)n hairpin with Pol β, which catalyze TNR expansion after hairpin incision [70].

## 7. Pols β and λ as Targets for Anticancer Chemotherapy

The concept of the DNA-repair interference as a potential adjuvant approach to overcome intrinsic or acquired tumor resistance is gaining substantial attention. Regarding the inhibition of DNA repair pathways, it is desirable to avoid harming normal cells. Therefore, preferably pathways that are alternatively activated just in cancer cells should be selectively targeted. Additionally, many proteins involved in repair pathways coordinate other pathways and functions, and their inhibition would lead to a catastrophe in cellular context. Thus, different inhibitors of key proteins in DNA repair have been developed [71,72].

Many studies have proposed a mutagenic role of deregulated specialized Pols in cancer. Research conducted by Albertella et al. [47] has offered the evidence that nearly 50% of different human tumors showed overexpression of one or more specialized Pols. The fact that Pols can help cancer cells tolerate DNA damage makes them interesting candidates for targeted therapy.

As summarized above, Pol β and λ play essential roles in DNA repair and DNA damage tolerance repair pathways. Abolishing the functions of these Pols appears then to be a powerful strategy in sensitizing tumor cells towards the conventional DNA damaging chemotherapy. 

Mounting evidence confirmed that overexpression of Pol β is a frequent event occurring in tumorigenesis. Pol β plays an important role in BER, an important drug-resistant determinant, due to the ability to rapidly and efficiently repair the DNA lesions induced by several chemotherapeutic agents [73]. Importantly, different human tumors are characterized by enhanced expression of Pol β, whose downregulation correlates with increased responsiveness to chemotherapy [74]. The ability of Pol β to bypass damaged DNA is largely exploited by cancer cells in order to boost their survival [75]. This is of particular clinical interest, as Pol β expression is increased in more than 30% of different human tumors [47], mostly ovarian, breast, uterus and prostate cancer. The same group confirmed the overexpression of Pol λ in 24% of various human tumors, which is comparable to the Pol β overexpression occurrence. Like Pol β, Pol λ has been proven to play a role in BER, as well [76,77]. Moreover, Pol λ physically and functionally interacts with the key components of NHEJ, the Ku antigen and the XRCC4/DNA ligase IV complex; indeed, Pol λ is central to the double-strand break repair pathway. The involvement of Pol λ in processes to safeguard the DNA integrity and ability to bypass some DNA lesions [14] contributing to the survival of cancer cells addresses the possibility to target this polymerase in the tumoral setting. Thus, different inhibitors for Pol β and λ are under study as potential anticancer agents.

### 7.1. Pol β Natural Inhibitors

The first attempts to discover the inhibitors of Pol β conducted by Mizushina et al. revealed that long chain fatty acids suppressed Pol activity [78]. Since the 1990s, several inhibitors of both polymerase [79,80,81,82,83,84,85,86,87,88,89] and lyase activity [90,91,92,93,94,95,96] of Pol β have been described. Of these, several natural compounds, like glycoglycerolipids [97], triterpenoids [81] and sulfolipids [98], are endowed with potent Pol β inhibitory activity; however, they unspecifically act on Pol α, as well. Other natural products, such as oleanolic acid, edgeworin, harbinatic acid and myristinin A, display a low micromolar inhibitory activity in biochemical assays and little toxicity [82,99,100]. Prunasin, a natural glucoside extracted from *Perilla frutescens* and *Artemisia vulgaris*, was demonstrated to be a specific Pol β inhibitor [101], since it did not act on mammalian Polα and TdT, plant Pols, HIV-1 Reverse Transcriptase nor on any prokaryotic Pol.

### 7.2. Pol β Synthetic Small Molecule Inhibitors

The deoxynucleotide analogue NSC-124854 identified by the group of Jaiswal et al. is an effective Pol β inhibitor, active against colorectal cancer cells with an EC_50_ value of 5.3 μM [102]. The small molecule methoxamine (MX) binds to and modifies AP sites, inhibiting lesion processing by the dRP-lyase activity of DNA polymerase β [103]. MX works in synergy with therapeutic alkylating agents (e.g., temozolomide (TMZ)) in order to potentiate their anti-tumoral potency in solid tumors [104,105], and has entered the phase I clinical trial process. The majority of the TMZ-induced DNA base adducts are removed by N-methylpurine DNA glycosylase (MPG), which initiates BER, leaving AP sites. Tang et al. [106] reported that potentiation of TMZ with MX, in glioma cells, is greatly enhanced by MPG overexpression. However, Pol β overexpression abrogated TMZ potentiation by MX, suggesting that cells proficient for BER readily repair AP sites in the presence of MX, and Pol β might be used to predict the effectiveness of MX-mediated potentiation of TMZ in cancer treatment. Potent Pol β inhibitors based on the rhodanine scaffold were recently discovered by the group of Strittmatter et al. [107]. Of 30 active compounds, 14 small-molecules have displayed specificity for Pol β. Additionally, several of the discovered compounds sensitized colorectal cancer cells towards DNA-damaging agents.

Noteworthy, Pol β activation and induction, which contribute to neuronal death, have been described in Alzheimer’s and Parkinson’s disease [108,109,110]. On that account, inhibitors of Pol β may provide a neuronal-specific activity, representing a successful strategy to combat this neurodegenerative disease. In 2015, the screening of more than 20,000 natural and millions of drug-like agents has been performed, leading to the identification of the 5-methoxyflavone endowed with the ability to inhibit DNA Pol β-mediated neurodegeneration without causing toxicity to normal neurons [111].

### 7.3. Pol λ Natural Inhibitors

Petasiphenol, a natural compound extracted from the Japanese plant *Petasites japonicus*, was proven to selectively inhibit Pol λ activity, but resulted in being ineffective towards the structurally-related Pol β, as well as towards replicative Pols [112]. The antioxidant and anti-inflammatory compound curcumin has been shown to inhibit Pol λ selectively and to suppress the growth of a human gastric cancer cell line [113]. Another potent natural compound that inhibits Pol λ activity belongs to the category of catechin derivatives and has been obtained from green tea *Camellia sinensis* [114]. However, this compound inhibited also Pol α and HIV-1 RT. The natural compounds from the class of tetralols, nodulisporol and nodulisporone, produced by a fungus (*Nodulisporium* sp.), were found to specifically inhibit Pol λ at the micromolar level [115]. In 2014, Mizushina et al. discovered that extracts from germinated soybean (*Glycine max* L.), composed mainly by glucosyls, specifically inhibited the activity of eukaryotic Pol λ and possessed anti-inflammatory properties [116].

### 7.4. Pol λ Synthetic Small Molecule Inhibitors

Specific methoxy-derivatives of resveratrol, a known antioxidant compound, were found to selectively inhibit Pol λ, but not the related Pol β and TdT [117].

In silico screening of more than 9000 compounds in order to discover molecular probes that selectively inhibit Pol λ yielded there novel classes of Pol λ inhibitors: rhodanines, carbohydrazides and the compounds with the 2,4-pentadione element [118]. Of these, rhodanines resulted in being the most potent Pol λ inhibitors. Further research on the rhodanine derivatives revealed ten compounds that proved to specifically target Pol λ [107] and synergistically potentiated the killing of colorectal cancer cells by DNA-damaging agents.

### 7.5. Dual Pols λ and β Natural Inhibitors

Some inhibitors identified in the past manifested dual activity on both Pol β and Pol λ. An example of such a natural inhibitor is solanapyrone A, which was discovered in 2002 by the group of Mizushina et al. [119]. Kimura et al. identified two azaphilones, kasanosins A and B, as specific Pol β and Pol λ inhibitors [120]. Other classes of natural compounds that inhibit specifically mammalian X family Pols (λ, β, TdT), with the strongest inhibitory activity towards Pol β, are represented by diallyl sulfides isolated from *Allium sativum* [121]. Interestingly, these compounds did not inhibit the activities of family A, B and Y Pols, as well as other DNA-metabolic enzymes, such as HIV-1 RT, T7 RNA polymerase and T4 polynucleotide kinase.

## 8. Conclusions and Perspectives

One of the most intriguing observations of the last decade, has been the realization that while only four Pols (α, δ, ξ and γ) are necessary and sufficient for the duplication of both nuclear and mitochondrial DNA, more than a dozen additional Pols are required in mammalian cells to ensure the maintenance of the genetic information. Biochemical, structural and genetic studies have revealed that these specialized Pols are endowed with special properties, which make them uniquely fit for a particular DNA repair event, it being either a special pathway or even a special DNA lesion. However, several of these enzymes have potentially overlapping roles, thus requiring careful regulation, in terms of expression levels, intracellular localization and timing of recruitment to a particular subcellular compartment. When such a tight regulation fails, these enzymes can be detrimental, rather than beneficial, to the cell, causing mutations and genetic instability.

This situation is well exemplified by the case of Pol β and λ. These enzymes play essential roles in many different repair pathways. Pol β is the main enzyme involved in BER, and it is essential during development, especially in the brain, as testified by the embryonic lethality of Pol β knockout mice. Pol λ, on the other hand, is not essential, at least for mouse development, since Pol λ knockout mice are viable and fertile. However, alterations of its levels have been clearly linked to various forms of DNA damage accumulation and genetic instability.

Both of these enzymes are thus fundamentally beneficial to the cell. However, in some special contexts, they can exert deleterious effects. For example, under unbalanced dNTP/rNTP pool ratios, both enzymes can incorporate rNMPs into DNA, causing mutations, DNA fragility and delaying BER of oxidized bases. During MMEJ of ends containing repetitive sequences, Pol β can also contribute to the deleterious expansion of CAG triplets. Finally, overexpression of both Pols has been clearly linked to tumorigenesis.

In this respect, such a dual aspect of Pol β and λ action, might be exploited for the better. In fact, understanding their mechanisms of (de)regulation is a key step towards their exploitation as potential antitumoral targets. For example, inhibition of Pol β has been shown to sensitize tumors towards conventional DNA damaging agents, while suppression of Pol λ has been shown to induce synthetic lethality when combined with Chk1 inhibitors.

Unfortunately, of the dozens of Pol λ and β inhibitors that have been described to date, both natural and small molecule compounds, only a small part is sufficiently selective and active in a non-toxic nanomolar range. In order to achieve improvements of the current treatment options, there is an imminent need to identify novel selective inhibitors targeting Pol λ and Pol β in tumor cells. The approach of the concurrent inhibition of DNA repair mechanisms and the use of systemic antitumoral therapy offers the rationale to potentiate selective tumor killing. However, knowledge of tumor background, comprehension of the altered DNA-repair mechanism, is essential in order to tailor the adequate antitumoral therapy. The research of novel promising agents to be exploited in anticancer therapy must thus be advanced, in order to optimize their selectivity, efficacy and reduce the mutagenic risk for healthy cells.

## Figures and Tables

**Table 1:** Polymerase β variants identified and involved in cancer.

Polymerase β Variant	Human Tumor Type
K289M [55]	Colorectal cancer
E288K [54]	
S229L [56]	
R152C [57]	
E295K	Gastric cancer
G231D	
L22P	
Y265C	
D160N [51]	
T889C [58]	
I260M [59]	Prostate cancer
P242R [60]	Evidence of chromosomal aberrations in human mammary cells
K167I [61]	Esophageal cancer

## References

[1] Khoronenkova S.V., Dianov G.L. (2011). The emerging role of mule and ARF in the regulation of base excision repair. FEBS Lett..

[2] Parsons J.L., Dianova I.I., Khoronenkova S.V., Edelmann M.J., Kessler B.M., Dianov G.L. (2011). Usp47 is a deubiquitylating enzyme that regulates base excision repair by controlling steady-state levels of DNA polymerase β. Mol. Cell.

[3] Frouin I., Toueille M., Ferrari E., Shevelev I., Hubscher U. (2005). Phosphorylation of human DNA polymerase λ by the cyclin-dependent kinase cdk2/cyclin a complex is modulated by its association with proliferating cell nuclear antigen. Nucl. Acids Res..

[4] Markkanen E., Hübscher U., van Loon B. (2012). Regulation of oxidative DNA damage repair: The adenine: 8-oxo-guanine problem. Cell Cycle.

[5] Zucca E., Bertoletti F., Wimmer U., Ferrari E., Mazzini G., Khoronenkova S., Grosse N., van Loon B., Dianov G., Hubscher U. (2013). Silencing of human DNA polymerase λ causes replication stress and is synthetically lethal with an impaired S phase checkpoint. Nucl. Acids Res..

[6] Cimprich K.A., Cortez D. (2008). ATR: An essential regulator of genome integrity. Nat. Rev. Mol. Cell. Biol..

[7] Moon A.F., Garcia-Diaz M., Batra V.K., Beard W.A., Bebenek K., Kunkel T.A., Wilson S.H., Pedersen L.C. (2007). The X family portrait: Structural insights into biological functions of X family polymerases. DNA Repair.

[8] Bebenek K., Pedersen L.C., Kunkel T.A. (2014). Structure-function studies of DNA polymerase λ. Biochemistry.

[9] Dianov G.L., Hübscher U. (2013). Mammalian base excision repair: The forgotten archangel. Nucl. Acids Res..

[10] Fortini P., Dogliotti E. (2007). Base damage and single-strand break repair: Mechanisms and functional significance of short- and long-patch repair subpathways. DNA Repair.

[11] Beard W.A., Wilson S.H. (2006). Structure and mechanism of DNA polymerase β. Chem. Rev..

[12] Gao Y., Katyal S., Lee Y., Zhao J., Rehg J.E., Russell H.R., McKinnon P.J. (2011). DNA ligase III is critical for mtDNA integrity but not Xrcc1-mediated nuclear DNA repair. Nature.

[13] Simsek D., Furda A., Gao Y., Artus J., Brunet E., Hadjantonakis A.-K., van Houten B., Shuman S., McKinnon P.J., Jasin M. (2011). Crucial roles for DNA ligase III in mitochondria but not in Xrcc1-dependent repair. Nature.

[14] Maga G., Villani G., Crespan E., Wimmer U., Ferrari E., Bertocci B., Hubscher U. (2007). 8-oxo-guanine bypass by human DNA polymerases in the presence of auxiliary proteins. Nature.

[15] Waters L.S., Minesinger B.K., Wiltrout M.E., D’Souza S., Woodruff R.V., Walker G.C. (2009). Eukaryotic translesion polymerases and their roles and regulation in DNA damage tolerance. Microbiol. Mol. Biol. Rev..

[16] Hübscher U., Maga G. (2011). DNA replication and repair bypass machines. Curr. Opin. Chem. Biol..

[17] Amoroso A., Crespan E., Wimmer U., Hubscher U., Maga G. (2008). DNA polymerases and oxidative damage: Friends or foes?. Curr. Mol. Pharmacol..

[18] Maga G., Crespan E., Wimmer U., van Loon B., Amoroso A., Mondello C., Belgiovine C., Ferrari E., Locatelli G., Villani G. (2008). Replication protein a and proliferating cell nuclear antigen coordinate DNA polymerase selection in 8-oxo-guanine repair. Proc. Natl. Acad. Sci. USA.

[19] Malyarchuk S., Castore R., Harrison L. (2008). DNA repair of clustered lesions in mammalian cells: Involvement of non-homologous end-joining. Nucl. Acids Res..

[20] Cunniffe S., O’Neill P., Greenberg M.M., Lomax M.E. (2014). Reduced repair capacity of a DNA clustered damage site comprised of 8-oxo-7,8-dihydro-2’-deoxyguanosine and 2-deoxyribonolactone results in an increased mutagenic potential of these lesions. Mutat. Res..

[21] Crespan E., Pasi E., Imoto S., Hübscher U., Greenberg M.M., Maga G. (2013). Human DNA polymerase β, but not λ, can bypass a 2-deoxyribonolactone lesion together with proliferating cell nuclear antigen. ACS Chem. Biol..

[22] Quiñones J.L., Thapar U., Yu K., Fang Q., Sobol R.W., Demple B. (2015). Enzyme mechanism-based, oxidative DNA–protein cross-links formed with DNA polymerase β in vivo. Proc. Natl. Acad. Sci. USA.

[23] Quiñones J.L., Demple B. (2016). When DNA repair goes wrong: BER-generated DNA-protein crosslinks to oxidative lesions. DNA Repair.

[24] Stevens A.J., Guan L., Bebenek K., Kunkel T.A., Greenberg M.M. (2013). DNA polymerase λ inactivation by oxidized abasic sites. Biochemistry.

[25] Blanca G., Villani G., Shevelev I., Ramadan K., Spadari S., Hübscher U., Maga G. (2004). Human DNA polymerases λ and β show different efficiencies of translesion DNA synthesis past abasic sites and alternative mechanisms for frameshift generation. Biochemistry.

[26] DeMott M.S., Beyret E., Wong D., Bales B.C., Hwang J.-T., Greenberg M.M., Demple B. (2002). Covalent trapping of human DNA polymerase β by the oxidative DNA lesion 2-deoxyribonolactone. J. Biol. Chem..

[27] Arian D., Hedayati M., Zhou H., Bilis Z., Chen K., DeWeese T.L., Greenberg M.M. (2014). Irreversible inhibition of DNA polymerase β by small molecule mimics of a DNA lesion. J. Am. Chem. Soc..

[28] Liu L., Rodriguez-Belmonte E.M., Mazloum N., Xie B., Lee M.Y. (2003). Identification of a novel protein, pdip38, that interacts with the p50 subunit of DNA polymerase δ and proliferating cell nuclear antigen. J. Biol. Chem..

[29] Tissier A., Janel-Bintz R., Coulon S., Klaile E., Kannouche P., Fuchs R.P., Cordonnier A.M. (2010). Crosstalk between replicative and translesional DNA polymerases: Pdip38 interacts directly with Polη. DNA Repair.

[30] Maga G., Crespan E., Markkanen E., Imhof R., Furrer A., Villani G., Hübscher U., van Loon B. (2013). DNA polymerase δ-interacting protein 2 is a processivity factor for DNA polymerase λ during 8-oxo-7,8-dihydroguanine bypass. Proc. Natl. Acad. Sci. USA.

[31] McElhinny S.A.N., Kumar D., Clark A.B., Watt D.L., Watts B.E., Lundström E.-B., Johansson E., Chabes A., Kunkel T.A. (2010). Genome instability due to ribonucleotide incorporation into DNA. Nat. Chem. Biol..

[32] Yao N.Y., Schroeder J.W., Yurieva O., Simmons L.A., O’Donnell M.E. (2013). Cost of rNTP/dNTP pool imbalance at the replication fork. Proc. Natl. Acad. Sci. USA.

[33] Clausen A.R., Murray M.S., Passer A.R., Pedersen L.C., Kunkel T.A. (2013). Structure–function analysis of ribonucleotide bypass by b family DNA replicases. Proc. Natl. Acad. Sci. USA.

[34] Reijns M.A., Rabe B., Rigby R.E., Mill P., Astell K.R., Lettice L.A., Boyle S., Leitch A., Keighren M., Kilanowski F. (2012). Enzymatic removal of ribonucleotides from DNA is essential for mammalian genome integrity and development. Cell.

[35] Brown J.A., Suo Z. (2011). Unlocking the sugar “steric gate” of DNA polymerases. Biochemistry.

[36] Nick McElhinny S.A., Ramsden D.A. (2003). Polymerase mu is a DNA-directed DNA/RNA polymerase. Mol. Cell. Biol..

[37] Martin M.J., Garcia-Ortiz M.V., Esteban V., Blanco L. (2013). Ribonucleotides and manganese ions improve non-homologous end joining by human polµ. Nucl. Acids Res..

[38] Crespan E., Furrer A., Rösinger M., Bertoletti F., Mentegari E., Chiapparini G., Imhof R., Ziegler N., Sturla S.J., Hübscher U. (2016). Impact of ribonucleotide incorporation by DNA polymerases β and λ on oxidative base excision repair. Nat. Commun..

[39] Gosavi R.A., Moon A.F., Kunkel T.A., Pedersen L.C., Bebenek K. (2012). The catalytic cycle for ribonucleotide incorporation by human DNA Pol λ. Nucl. Acids Res..

[40] Cilli P., Minoprio A., Bossa C., Bignami M., Mazzei F. (2015). Formation and repair of mismatches containing ribonucleotides and oxidized bases at repeated DNA sequences. J. Biol. Chem..

[41] Frit P., Barboule N., Yuan Y., Gomez D., Calsou P. (2014). Alternative end-joining pathway(s): Bricolage at DNA breaks. DNA Repair.

[42] Lieber M.R. (2010). The mechanism of double-strand DNA break repair by the nonhomologous DNA end joining pathway. Annu. Rev. Biochem..

[43] Crespan E., Czabany T., Maga G., Hübscher U. (2012). Microhomology-mediated DNA strand annealing and elongation by human DNA polymerases λ and β on normal and repetitive DNA sequences. Nucl. Acids Res..

[44] Barone F., McCulloch S.D., Macpherson P., Maga G., Yamada M., Nohmi T., Minoprio A., Mazzei F., Kunkel T.A., Karran P. (2007). Replication of 2-hydroxyadenine-containing DNA and recognition by human mutsalpha. DNA Repair.

[45] Marechal A., Zou L. (2013). DNA damage sensing by the ATM and ATR kinases. Cold Spring Harb. Perspect. Biol..

[46] Kelly T.J., Brown G.W. (2000). Regulation of chromosome replication. Annu. Rev. Biochem..

[47] Albertella M.R., Lau A., O’Connor M.J. (2005). The overexpression of specialized DNA polymerases in cancer. DNA Repair.

[48] Capp J.-P., Boudsocq F., Bergoglio V., Trouche D., Cazaux C., Blanco L., Hoffmann J.-S., Canitrot Y. (2010). The R438W polymorphism of human DNA polymerase λ triggers cellular sensitivity to camptothecin by compromising the homologous recombination repair pathway. Carcinogenesis.

[49] Terrados G., Capp J.-P., Canitrot Y., García-Díaz M., Bebenek K., Kirchhoff T., Villanueva A., Boudsocq F., Bergoglio V., Cazaux C. (2009). Characterization of a natural mutator variant of human DNA polymerase λ which promotes chromosomal instability by compromising NHEJ. PLoS ONE.

[50] Fang Q., Inanc B., Schamus S., Wang X.H., Wei L., Brown A.R., Svilar D., Sugrue K.F., Goellner E.M., Zeng X. (2014). HSP90 regulates DNA repair via the interaction between XRCC1 and DNA polymerase β. Nat. Commun..

[51] Iwanaga A., Ouchida M., Miyazaki K., Hori K., Mukai T. (1999). Functional mutation of DNA polymerase β found in human gastric cancer--inability of the base excision repair in vitro. Mutat. Res..

[52] Dobashi Y., Shuin T., Tsuruga H., Uemura H., Torigoe S., Kubota Y. (1994). DNA polymerase β gene mutation in human prostate cancer. Cancer Res..

[53] Starcevic D., Dalal S., Sweasy J.B. (2004). Is there a link between DNA polymerase β and cancer?. Cell Cycle.

[54] Donigan K.A., Sun K.W., Nemec A.A., Murphy D.L., Cong X., Northrup V., Zelterman D., Sweasy J.B. (2012). Human polb gene is mutated in high percentage of colorectal tumors. J. Biol. Chem..

[55] Lang T., Maitra M., Starcevic D., Li S.X., Sweasy J.B. (2004). A DNA polymerase β mutant from colon cancer cells induces mutations. Proc. Natl. Acad. Sci. USA.

[56] Nemec A.A., Murphy D.L., Donigan K.A., Sweasy J.B. (2014). The s229l colon tumor-associated variant of DNA polymerase β induces cellular transformation as a result of decreased polymerization efficiency. J. Biol. Chem..

[57] Zhou T., Pan F., Cao Y., Han Y., Zhao J., Sun H., Zhou X., Wu X., He L., Hu Z. (2016). R152c DNA pol beta mutation impairs base excision repair and induces cellular transformation. Oncotarget.

[58] Tan X., Wang H., Luo G., Ren S., Li W., Cui J., Gill H.S., Fu S.W., Lu Y. (2015). Clinical significance of a point mutation in DNA polymerase beta (polb) gene in gastric cancer. Int. J. Biol. Sci..

[59] Dalal S., Hile S., Eckert K.A., Sun K.-W., Starcevic D., Sweasy J.B. (2005). Prostate-cancer-associated i260m variant of DNA polymerase β is a sequence-specific mutator. Biochemistry.

[60] Yamtich J., Nemec A.A., Keh A., Sweasy J.B. (2012). A germline polymorphism of DNA polymerase beta induces genomic instability and cellular transformation. PLoS Genet..

[61] Wang Y., Zang W., Du Y., Chen X., Zhao G. (2015). The k167i variant of DNA polymerase β that is found in esophageal carcinoma patients impairs polymerase activity and BER. Sci. Rep..

[62] Ray S., Menezes M.R., Senejani A., Sweasy J.B. (2013). Cellular roles of DNA polymerase β. Yale J. Biol. Med..

[63] Louat T., Servant L., Rols M.P., Bieth A., Teissie J., Hoffmann J.S., Cazaux C. (2001). Antitumor activity of 2′,3′-dideoxycytidine nucleotide analog against tumors up-regulating DNA polymerase β. Mol. Pharmacol..

[64] Bergoglio V., Frechet M., Philippe M., Bieth A., Mercier P., Morello D., Lacroix-Tricki M., Delsol G., Hoffmann J.S., Cazaux C. (2004). Evidence of finely tuned expression of DNA polymerase β in vivo using transgenic mice. FEBS Lett..

[65] Frechet M., Canitrot Y., Cazaux C., Hoffmann J.S. (2001). DNA polymerase β imbalance increases apoptosis and mutagenesis induced by oxidative stress. FEBS Lett..

[66] Frechet M., Canitrot Y., Bieth A., Dogliotti E., Cazaux C., Hoffmann J.S. (2002). Deregulated DNA polymerase β strengthens ionizing radiation-induced nucleotidic and chromosomal instabilities. Oncogene.

[67] Fotiadou P., Henegariu O., Sweasy J.B. (2004). DNA polymerase β interacts with TRF2 and induces telomere dysfunction in a murine mammary cell line. Cancer Res..

[68] Goula A.-V., Merienne K. (2013). Abnormal base excision repair at trinucleotide repeats associated with diseases: A tissue-selective mechanism. Genes.

[69] Liu Y., Wilson S.H. (2012). DNA base excision repair: A mechanism of trinucleotide repeat expansion. Trends Biochem. Sci..

[70] Guo J., Gu L., Leffak M., Li G.-M. (2016). MutSβ promotes trinucleotide repeat expansion by recruiting DNA polymerase β to nascent (CAG) n or (CTG) n hairpins for error-prone DNA synthesis. Cell Res..

[71] Ding J., Miao Z.H., Meng L.H., Geng M.Y. (2006). Emerging cancer therapeutic opportunities target DNA-repair systems. Trends Pharmacol. Sci..

[72] Madhusudan S., Hickson I.D. (2005). DNA repair inhibition: A selective tumour targeting strategy. Trends Mol. Med..

[73] Liu L., Nakatsuru Y., Gerson S.L. (2002). Base excision repair as a therapeutic target in colon cancer. Clin. Cancer Res..

[74] Canitrot Y., Cazaux C., Frechet M., Bouayadi K., Lesca C., Salles B., Hoffmann J.S. (1998). Overexpression of DNA polymerase β in cell results in a mutator phenotype and a decreased sensitivity to anticancer drugs. Proc. Natl. Acad. Sci. USA.

[75] Lange S.S., Takata K., Wood R.D. (2011). DNA polymerases and cancer. Nat. Rev. Cancer.

[76] Lebedeva N.A., Rechkunova N.I., Dezhurov S.V., Khodyreva S.N., Favre A., Blanco L., Lavrik O.I. (2005). Comparison of functional properties of mammalian DNA polymerase λ and DNA polymerase β in reactions of DNA synthesis related to DNA repair. Biochim. Biophys. Acta.

[77] Fiala K.A., Abdel-Gawad W., Suo Z. (2004). Pre-steady-state kinetic studies of the fidelity and mechanism of polymerization catalyzed by truncated human DNA polymerase λ. Biochemistry.

[78] Mizushina Y., Tanaka N., Yagi H., Kurosawa T., Onoue M., Seto H., Horie T., Aoyagi N., Yamaoka M., Matsukage A. (1996). Fatty acids selectively inhibit eukaryotic DNA polymerase activities in vitro. Biochim. Biophys. Acta.

[79] Ono K., Nakane H., Fukushima M., Chermann J.C., Barre-Sinoussi F. (1990). Differential inhibitory effects of various flavonoids on the activities of reverse transcriptase and cellular DNA and RNA polymerases. Eur. J. Biochem..

[80] Chen J., Zhang Y.H., Wang L.K., Sucheck S.J., Snow A.M., Hecht S.M. (1998). Inhibitors of DNA polymerase β from schoepfia californica. Chem. Commun..

[81] Tanaka N., Kitamura A., Mizushina Y., Sugawara F., Sakaguchi K. (1998). Fomitellic acids, triterpenoid inhibitors of eukaryotic DNA polymerases from a basidiomycete, *Fomitella fraxinea*. J. Nat. Prod..

[82] Deng J.Z., Sun Di-An A., Starck S.R., Hecht S.M., Cerny R.L., Engen J.R. (1999). Chrysochlamic acid, a new diterpenoid-substituted quinol from chrysochlamys ulei that inhibits DNA polymerase β. J. Chem. Soc. Perkin Trans. 1.

[83] Sun D.A., Deng J.Z., Starck S.R., Hecht S.M. (1999). Mispyric acid, a new monocyclic triterpenoid with a novel skeleton from *Mischocarpus pyriformis* that inhibits DNA polymerase β. J. Am. Chem. Soc..

[84] Sun D.A., Starck S.R., Locke E.P., Hecht S.M. (1999). DNA polymerase β inhibitors from *Sandoricum koetjape*. J. Nat. Prod..

[85] Deng J.Z., Starck S.R., Hecht S.M. (1999). DNA polymerase β inhibitors from *Baeckea gunniana*. J. Nat. Prod..

[86] Deng J.Z., Starck S.R., Hecht S.M., Ijames C.F., Hemling M.E. (1999). Harbinatic acid, a novel and potent DNA polymerase β inhibitor from *Hardwickia binata*. J. Nat. Prod..

[87] Ma J., Starck S.R., Hecht S.M. (1999). DNA polymerase β inhibitors from *Tetracera boiviniana*. J. Nat. Prod..

[88] Deng J.Z., Starck S.R., Hecht S.M. (2000). Pentacyclic triterpenoids from freziera sp. that inhibit DNA polymerase β. Bioorg. Med. Chem..

[89] Deng J.Z., Starck S.R., Sun D.A., Sabat M., Hecht S.M. (2000). A new 7,8-euphadien-type triterpenoid from *Brackenridgea nitida* and *Bleasdalea bleasdalei* that inhibits DNA polymerase β. J. Nat. Prod..

[90] Hecht S.M. (2003). Inhibitors of the lyase activity of DNA polymerase β. Pharm. Biol..

[91] Prakash Chaturvedula V.S., Gao Z., Hecht S.M., Jones S.H., Kingston D.G. (2003). A new acylated oleanane triterpenoid from *Couepia polyandra* that inhibits the lyase activity of DNA polymerase β. J. Nat. Prod..

[92] Li S.S., Gao Z., Feng X., Jones S.H., Hecht S.M. (2004). Plant sterols as selective DNA polymerase β lyase inhibitors and potentiators of *Bleomycin cytotoxicity*. Bioorg. Med. Chem..

[93] Li S.S., Gao Z., Feng X., Hecht S.M. (2004). Biscoumarin derivatives from edgeworthia gardneri that inhibit the lyase activity of DNA polymerase β. J. Nat. Prod..

[94] Prakash Chaturvedula V.S., Hecht S.M., Gao Z., Jones S.H., Feng X., Kingston D.G. (2004). New neolignans that inhibit DNA polymerase β lyase. J. Nat. Prod..

[95] Chaturvedula V.S., Zhou B.N., Gao Z., Thomas S.J., Hecht S.M., Kingston D.G. (2004). New lupane triterpenoids from *Solidago canadensis* that inhibit the lyase activity of DNA polymerase β. Bioorg. Med. Chem..

[96] Feng X., Gao Z., Li S., Jones S.H., Hecht S.M. (2004). DNA polymerase β lyase inhibitors from *Maytenus putterlickoides*. J. Nat. Prod..

[97] Ogawa A., Murate T., Izuta S., Takemura M., Furuta K., Kobayashi J., Kamikawa T., Nimura Y., Yoshida S. (1998). Sulfated glycoglycerolipid from Archaebacterium inhibits eukaryotic DNA polymerase alpha, β and retroviral reverse transcriptase and affects methyl methanesulfonate cytotoxicity. Int. J. Cancer.

[98] Mizushina Y., Watanabe I., Ohta K., Takemura M., Sahara H., Takahashi N., Gasa S., Sugawara F., Matsukage A., Yoshida S. (1998). Studies on inhibitors of mammalian DNA polymerase α and β: Sulfolipids from a pteridophyte, *Athyrium niponicum*. Biochem. Pharmacol..

[99] Gao Z., Maloney D.J., Dedkova L.M., Hecht S.M. (2008). Inhibitors of DNA polymerase β: Activity and mechanism. Bioorg. Med. Chem..

[100] Maloney D.J., Deng J.Z., Starck S.R., Gao Z., Hecht S.M. (2005). (+)-myristinin A, a naturally occurring DNA polymerase β inhibitor and potent DNA-damaging agent. J. Am. Chem. Soc..

[101] Mizushina Y., Takahashi N., Ogawa A., Tsurugaya K., Koshino H., Takemura M., Yoshida S., Matsukage A., Sugawara F., Sakaguchi K. (1999). The cyanogenic glucoside, prunasin (D-mandelonitrile-β-D-glucoside), is a novel inhibitor of DNA polymerase β. J. Biochem..

[102] Jaiswal A.S., Banerjee S., Aneja R., Sarkar F.H., Ostrov D.A., Narayan S. (2011). DNA polymerase β as a novel target for chemotherapeutic intervention of colorectal cancer. PLoS ONE.

[103] Horton J.K., Prasad R., Hou E., Wilson S.H. (2000). Protection against methylation-induced cytotoxicity by DNA polymerase β-dependent long patch base excision repair. J. Biol. Chem..

[104] Taverna P., Liu L., Hwang H.S., Hanson A.J., Kinsella T.J., Gerson S.L. (2001). Methoxyamine potentiates DNA single strand breaks and double strand breaks induced by temozolomide in colon cancer cells. Mutat. Res..

[105] Fishel M.L., He Y., Smith M.L., Kelley M.R. (2007). Manipulation of base excision repair to sensitize ovarian cancer cells to alkylating agent temozolomide. Clin. Cancer Res..

[106] Tang J.B., Svilar D., Trivedi R.N., Wang X.H., Goellner E.M., Moore B., Hamilton R.L., Banze L.A., Brown A.R., Sobol R.W. (2011). N-methylpurine DNA glycosylase and DNA polymerase β modulate BER inhibitor potentiation of glioma cells to temozolomide. Neuro-oncology.

[107] Strittmatter T., Brockmann A., Pott M., Hantusch A., Brunner T., Marx A. (2014). Expanding the scope of human DNA polymerase λ and β inhibitors. ACS Chem. Biol..

[108] Copani A., Sortino M.A., Caricasole A., Chiechio S., Chisari M., Battaglia G., Giuffrida-Stella A.M., Vancheri C., Nicoletti F. (2002). Erratic expression of DNA polymerases by β-amyloid causes neuronal death. FASEB J..

[109] Copani A., Hoozemans J.J., Caraci F., Calafiore M., Van Haastert E.S., Veerhuis R., Rozemuller A.J., Aronica E., Sortino M.A., Nicoletti F. (2006). DNA polymerase-β is expressed early in neurons of alzheimer’s disease brain and is loaded into DNA replication forks in neurons challenged with β-amyloid. J. Neurosci..

[110] Zhang Z., Cao X., Xiong N., Wang H., Huang J., Sun S., Liang Z., Wang T. (2010). DNA polymerase-β is required for 1-methyl-4-phenylpyridinium-induced apoptotic death in neurons. Apoptosis.

[111] Merlo S., Basile L., Giuffrida M.L., Sortino M.A., Guccione S., Copani A. (2015). Identification of 5-methoxyflavone as a novel DNA polymerase-β inhibitor and neuroprotective agent against β-amyloid toxicity. J. Nat. Prod..

[112] Mizushina Y., Kamisuki S., Kasai N., Ishidoh T., Shimazaki N., Takemura M., Asahara H., Linn S., Yoshida S., Koiwai O. (2002). Petasiphenol: A DNA polymerase λ inhibitor. Biochemistry.

[113] Mizushina Y., Hirota M., Murakami C., Ishidoh T., Kamisuki S., Shimazaki N., Takemura M., Perpelescu M., Suzuki M., Yoshida H. (2003). Some anti-chronic inflammatory compounds are DNA polymerase λ-specific inhibitors. Biochem. Pharmacol..

[114] Mizushina Y., Saito A., Tanaka A., Nakajima N., Kuriyama I., Takemura M., Takeuchi T., Sugawara F., Yoshida H. (2005). Structural analysis of catechin derivatives as mammalian DNA polymerase inhibitors. Biochem. Biophys. Res. Commun..

[115] Kamisuki S., Ishimaru C., Onoda K., Kuriyama I., Ida N., Sugawara F., Yoshida H., Mizushina Y. (2007). Nodulisporol and nodulisporone, novel specific inhibitors of human DNA polymerase λ from a fungus, *Nodulisporium* sp.. Bioorg. Med. Chem..

[116] Mizushina Y., Kuriyama I., Yoshida H. (2014). Inhibition of DNA polymerase λ and associated inflammatory activities of extracts from steamed germinated soybeans. Food Funct..

[117] Locatelli G.A., Savio M., Forti L., Shevelev I., Ramadan K., Stivala L.A., Vannini V., Hubscher U., Spadari S., Maga G. (2005). Inhibition of mammalian DNA polymerases by resveratrol: Mechanism and structural determinants. Biochem. J..

[118] Strittmatter T., Bareth B., Immel T.A., Huhn T., Mayer T.U., Marx A. (2011). Small molecule inhibitors of human DNA polymerase λ. ACS Chem. Biol..

[119] Mizushina Y., Kamisuki S., Kasai N., Shimazaki N., Takemura M., Asahara H., Linn S., Yoshida S., Matsukage A., Koiwai O. (2002). A plant phytotoxin, solanapyrone A, is an inhibitor of DNA polymerase β and λ. J. Biol. Chem..

[120] Kimura T., Nishida M., Kuramochi K., Sugawara F., Yoshida H., Mizushina Y. (2008). Novel azaphilones, kasanosins A and B, which are specific inhibitors of eukaryotic DNA polymerases β and λ from talaromyces sp.. Bioorg. Med. Chem..

[121] Nishida M., Hada T., Kuramochi K., Yoshida H., Yonezawa Y., Kuriyama I., Sugawara F., Yoshida H., Mizushina Y. (2008). Diallyl sulfides: Selective inhibitors of family X DNA polymerases from garlic (*Allium sativum* L.). Food Chem..

